# Diseases of the Nervous System

**Published:** 1901-06

**Authors:** 


DISEASES OF THE NERVOUS SYSTEM. A text-book for students and practitioners of medicine. By H OPPENHEIM, M. D., Professor at the University of Berlin. Authorised translation by Edward E. Mayer, A. M., M. D., Pittsburg. First American from the second revised and enlarged German edition. Large 8vo., pp. 899. With 293 illustrations. Philadelphia and London: J. B. Lippincott Co. 1900.

Among the younger German neurologists none has acquired more popularity than the able assistant of the late Professor Westphal. The Charity Hospital at Berlin affords opportunities unexcelled on the continent, and in the midst of such surroundings the author of this excellent work had his workshop. Careful, painstaking examinations, and logical deductions, characterise Oppenheim's work whether in the hospital ward, laboratory, or clinic room and these characteristics have borne fruit as evidenced by the success which has attended his work on nervous diseases. Oppenheim has striven to make this work useful to the practitioner and has laid particular stress on diagnosis, symptomatology and treatment, while the pathological anatomy underlying the different diseases has been curtailed in so much that only what was necessary to shed light upon the course of a disease, or to pave the way for a diagnosis is given.

The author divides his work into two parts—the general part treating of the methods of examination, general symptomatology and objective examination; and Part II., or special part, treating of the various diseases. In speaking of the tendon reflexes, the author deprecates the prevalent habit of calling an absent reflex, Westphal's sign. It is customary in this country at least to call it a Westphal sign—*only* when occurring in locomotor ataxia, and not when occurring in infantile paralysis, or toxic neuritis, for instance. The chapter on electro-diagnosis, is fully described and presents a clear exposition of this important aid in making critical examinations.

Under Part II., the author takes up the diseases of the spinal cord, peripheral nerves, brain, the neuroses, troplo and angioneurosis, and toxic conditions with predominating nervous symptoms. In describing these various diseases, the author has given due consideration to other observers, but hardly a chapter in which he himself has not made notable additions to our knowledge on the subject. Myelitis, locomotor ataxia, the traumatic neuroses, brain tumors, the diseases of the pons and medulla, Oppenheim's discoveries stand out in good light In this edition reference has been made to the *x* ray in diagnosticating disorders of the vertebral column, and brain, but in a very conservative manner.

The illustrations are many, but some are not as clear as in the German edition. On the whole, no better text-book on neurology exists in the German language than that of Oppenheim's. The translation is even, accurate and a credit to the American edition. Binding, paper and printing are of Lippincott's best. W. C. K.

